# Mechanical Regulation of Limb Bud Formation

**DOI:** 10.3390/cells11030420

**Published:** 2022-01-26

**Authors:** Yvenn Sermeus, Jef Vangheel, Liesbet Geris, Bart Smeets, Przemko Tylzanowski

**Affiliations:** 1 MeBioS, KU Leuven, 3000 Leuven, Belgium; yvenn.sermeus@kuleuven.be (Y.S.); jef.vangheel@kuleuven.be (J.V.); Bart.Smeets@kuleuven.be (B.S.); 2 Prometheus, Division of Skeletal Tissue Engineering, KU Leuven, 3000 Leuven, Belgium; liesbet.geris@kuleuven.be; 3 GIGA In Silico Medicine, Université de Liège, 4000 Liège, Belgium; 4 SBE, Department of Development and Regeneration, KU Leuven, Herestraat 49, 3000 Leuven, Belgium; 5 Laboratory of Molecular Genetics, Department of Biomedical Sciences, Medical University of Lublin, Chodzki 1, 20-093 Lublin, Poland

**Keywords:** limb bud, morphogenesis, developmental mechanics

## Abstract

Early limb bud development has been of considerable interest for the study of embryological development and especially morphogenesis. The focus has long been on biochemical signalling and less on cell biomechanics and mechanobiology. However, their importance cannot be understated since tissue shape changes are ultimately controlled by active forces and bulk tissue rheological properties that in turn depend on cell–cell interactions as well as extracellular matrix composition. Moreover, the feedback between gene regulation and the biomechanical environment is still poorly understood. In recent years, novel experimental techniques and computational models have reinvigorated research on this biomechanical and mechanobiological side of embryological development. In this review, we consider three stages of early limb development, namely: outgrowth, elongation, and condensation. For each of these stages, we summarize basic biological regulation and examine the role of cellular and tissue mechanics in the morphogenetic process.

## 1. Introduction

Morphogenesis involves the coordinated growth and movement of cells to produce shapes and patterns that underlie the spatial organization of tissues and organs. It has long been known that cell and tissue mechanics play a crucial role in this process. However, for many years, conceptual models have mainly focused on the integration of chemical signaling and molecular genetics, rather than descriptions that include developmental mechanics. One of the main challenges for mechanical descriptions is that they are necessarily quantitative and that they need to account for context-dependent behavior, thereby necessitating advanced in situ characterization tools. Nonetheless, in recent years, novel experimental techniques and computational models have reinvigorated biomechanical and mechanobiological research, as well as its link to biochemistry [1].

**Figure 1:**
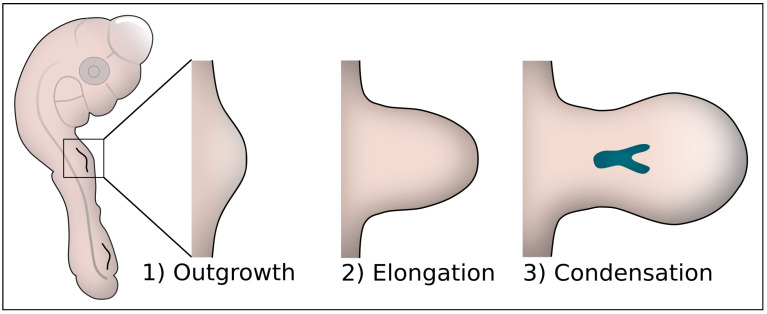
The three stages of early limb bud development. (**1**) In the initial outgrowth stage, a bulge is formed in the lateral plate mesoderm. (**2**) This bulge elongates in the proximodistal direction and widens to form a paddle shape. (**3**) Simultaneously with the elongation, condensing zones are formed which will eventually form musculoskeletal elements of the limb.

The most popular embryological model of complex morphogenesis is the developing vertebrate limb. Early limb bud formation occurs in three stages, initiation, elongation and condensation, as shown in Figure 1. In the first stage, the initial outgrowth/protrusion from the lateral plate mesoderm is formed. This initiation is an apparently isotropic rounding-up of the mesenchyme in the lateral plate mesoderm. It differs from the subsequent elongation where the breaking of symmetry occurs and the limb bud actively elongates [2]. In parallel with elongation, condensations are formed, which designate the prospective skeletal elements of the limb. Whereas chemical signaling and the role of Positional Information (PI) or Reaction Diffusion (RD) [3,4] have been studied extensively for many years [5,6], the physical mechanisms that govern limb bud morphogenesis only gained interest recently.

Much of the recent progress in the physics of limb development can be attributed to novel measuring techniques, focusing on mechanical aspects of tissues and cells, as well as computational models complementing these experiments. In this review paper, we compile recent findings on the role of mechanical and geometric regulation during the three stages of early limb bud development. For each of these stages, the basic biological regulation is first summarized and subsequently integrated with recent studies on the role of mechanical and biophysical control of the morphogenetic process.

## 2. Biophysics of Outgrowth

**Figure 2:**
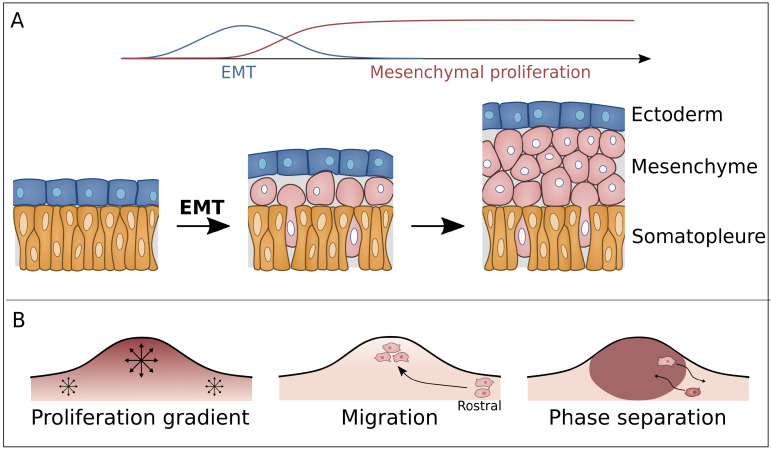
(**A**) Prior to limb bud outgrowth, the polarized and columnar somatopleural cells in the lateral plate mesoderm, shown in yellow, undergo an EMT transition followed by mesenchymal proliferation. (**B**) Simultaneously with this mesenchymal proliferation the limb bud outgrowth is formed through different possible mechanisms. (1) A proliferation gradient with higher proliferation rates towards the developing apical ectodermal ridge, (2) migration of mesenchymal cells from flanking regions into the limb field and (3) phase separation of the more fluidized limb cells from the flanking cells.

The limb bud is formed from the lateral plate mesoderm consisting out of a single layer of mesodermal cells that line the coelomic cavity and are covered by a single layer of ectodermal cells. Limb formation is initialized by an epithelial-to-mesenchymal transition (EMT) in the limb forming mesoderm (Figure 2A), induced by FGF8 [7,8,9,10]. These newly formed mesenchymal cells will bulge out of the body wall to form the limb bud. However, the biophysical mechanisms driving this process are still unclear [7,8].

### 2.1. Proliferative Outgrowth & Migration

The first proposed mechanism, called ‘growth-based morphogenesis’, hypothesizes that the outgrowth is driven by a higher proliferative rate in the limb-forming region compared to the flanking mesoderm (Figure 2B, left panel) [7,11,12]. Computational models have demonstrated how outgrowth and even elongation could indeed occur through this process [13]. However, computational models show that realistic proliferation rates do not lead to elongation [14]. Additionally, proliferation in the lateral plate mesoderm appears to be similar in both limb-forming and trunk-forming tissue during the first stages of outgrowth, making it unconvincing that growth-based morphogenesis alone can adequately explain limb initiation [7,11]. To address these inconsistencies, directional cell movement was proposed as a mechanism for outgrowth. In experiments that involve lineage tracing and live imaging in transgenic mice and chick, the occurrence of directional movement of cells in the lateral plate mesoderm has been observed [2]. Specifically, cells were found to migrate into the limb field in a rostral-to-caudal direction (Figure 2B, middle panel). This directional movement is likely to be regulated by WNT5a [2,7,15]. Additionally, these observations suggest different mechanisms for limb bud initiation and elongation, the former being dependent on the lateral plate [2].

### 2.2. Unjamming Transition & Phase Separation

During the epithelial-to-mesenchymal transition (EMT), limb-forming mesoderm cells appear more cohesive compared to trunk-forming epithelial-like cells [16]. Based on the differential adhesion hypothesis (DAH) and the differential interfacial tension hypothesis (DITH) [17,18], it has been proposed that cohesive limb-forming cells phase separate from trunk-forming cells, similar to immiscible liquids attaining a state of minimal free energy (Figure 2B, right panel) [19]. However, according to DITH, the newly phase separated limb bud cells will become engulfed by the trunk forming region as opposed to forming the observed bulging from the body wall [19]. Damon et al. suggested that an active mechanical response of the flank tissue could inhibit complete engulfment. According to this theory, the pressure arising due to engulfment of the limb bud cells triggers a disproportional response from the flanking tissue resulting in an outward net force [16]. Alternatively, the presence of the ectoderm could be sufficient to inhibit engulfment. In this case contact with the ectoderm lowers the surface tension of both tissue types to a similar level, thereby decreasing the driving force of engulfment [20]. Another hypothesis considers the EMT as an unjamming phase transition induced by FGF. In the jammed state, cell displacement is limited by interactions with neighboring cells, effectively locking them in position, resembling a solid-like material. In contrast, in the unjammed state, cells can frequently exchange neighbors, resembling a fluid-like material. The jammed state of the trunk-forming mesoderm would prevent engulfment as the minimal free energy state will not be obtained in the jammed material [21]. This hypothesis is compatible with theoretical descriptions of tissues based on vertex models, which show that an increase in cell–cell adhesion may induce liquid-like behavior [22,23,24]. This phase transition is controlled by FGF signaling, which guides the unjamming transition by increasing cell–cell adhesion/lowering cell interfacial tension, explaining the increase in tissue cohesiveness. Alternatively, an increase in cell motility as observed in zebrafish and chicken tail elongation can also induce an unjamming transition [25,26].

## 3. Mechanisms of Extension and Shape Formation

Once the limb bud is established, growth becomes distinctly anisotropic as the limb bud elongates and widens distally to establish the characteristic paddle shape. This process has been extensively studied as an example of robust non-isotropic growth. As with the initial outgrowth, growth-based morphogenesis started out as the leading hypothesis [27]. This theory is supported by the observation that FGFs secreted by the apical ectodermal ridge (AER) create a proliferation gradient [28,29]. However, recently, more accurate cell cycle times were mapped on 2D limb bud sections using a double labeling technique with IddU and BrdU. These experiments showed that between HH23-27, cell cycle times vary between 11 and 25 h. Additionally, a 3D Finite Element Model (FEM, see Box 1) showed that the difference in proliferative rate between distal and proximal regions is insufficient to model the elongating shape of the limb bud [14].

### 3.1. Directed Cell Division

Since the spatio-temporal profile of proliferation rates alone does not reproduce the observed limb elongation, other cellular mechanisms must be considered. It has been shown that after initiation, the elongation of cells redirects towards the nearest ectoderm [2]. This oriented behavior is triggered by WNT5a which regulates planar cell polarity (PCP) [15,30]. At both ends of the elongated cells, highly dynamic filopodia are present [14,30]. The presence and directionality of these filopodia is crucial for elongation, evidenced by the reduced limb length of WNT5a−/− mutants [31]. These filopodia play a role in chemical signaling [32] but their mechanical role is still unclear.

**Figure 3:**
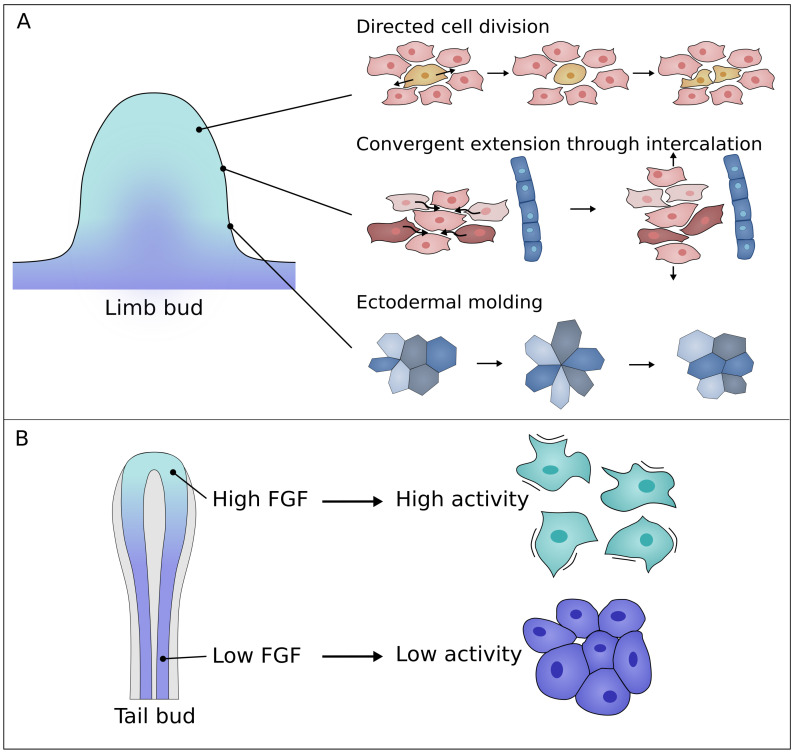
(**A**) Illustration of the main current hypotheses explaining limb bud elongation. (1) Directed division of mesenchymal cells. (2) Intercalation of mesenchymal cells. (3) Formation and resolution of rosettes in the ectoderm a form of ectodermal molding. (**B**) Tail bud elongation: Cell motility in the tail bud is controlled by an FGF gradient. Highly motile cells at the tail end are sparser compared to less motile cells at lower FGF concentrations.

Cell elongation affects the orientation of cell division: cells divide along the long axis; this is known as Hertwig’s rule [33]. Such an oriented division could be responsible for tissue scale elongation in the same direction, as has been observed in the drosophila wing disc or zebrafish neural tube [34,35]. Indeed, in the early limb bud, the predominant PD orientation of cells is consistent with the profile of limb elongation. However, at later stages, the direction of cell divisions is biased perpendicular to the PD axis in large portions of the limb bud (Figure 3A) [30,36]. Here, oriented cell division would be expected to widen the limb and not elongate it, assuming cell rearrangements are limited. Moreover, it was found that inhibition of cell division, using trichostatin to prevent cells from entering the S phase, does not affect anisotropy in the limb bud. This would suggest that the contribution of oriented division is minor in the process of limb bud elongation [37]. Furthermore, in ovo imaging of cell division revealed that daughter cells occupy a similar space as the mother cell and hence do not elongate the tissue [36].

### 3.2. Collective Migration

Once the limb bud outgrowth is established, migration from the lateral plate into the limb stops [2]. Yet, it has been proposed that the migratory movement of cells within the limb is a source of anisotropy. Chemo-attractant properties of FGF4 and WNT5a were revealed using implanted micro-carrier beads [30,38]. It was suggested that the high concentration of these morphogens near the AER causes cell elongation towards the AER [38]. However, in other experiments, the cells were found to be oriented towards the nearest ectoderm and not the AER leading to a general cell orientation perpendicular to the PD-axis [14,36]. Furthermore, cells within the tissue appear to be at rest for the greater part of their cell cycle, despite active extension and retraction of filopodia. Cells switch to migratory behavior for relatively short periods and most likely do so immediately after division [36]. Recently, haptotaxis and durotaxis, cell migration on a gradient of adhesion and stiffness, respectively, have been hypothesized as a driver for cell migration in the limb bud [39]. A gradient with high stiffness in the proximal central region of the limb bud and lower stiffness near the AER can cause the cells to deform as if elongating towards the nearest ectoderm. However, although durotaxis has been observed in vitro, no conclusive evidence exists that this mechanism plays an important role during in vivo limb development [39].

### 3.3. Intercalation

More recently, convergent extension (CE) by directed cell intercalations was proposed as a mechanism for limb bud elongation. CE has been observed during Xenopus and chick gastrulation [40,41,42,43]. In CE, cellular rearrangements cause the tissue to narrow in one planar axis while lengthening perpendicular to this axis (Figure 3A) [44]. In the limb bud, this theory is supported by the orientation of mesenchymal cells which is mainly perpendicular to the PD axis [30]. Different computational models illustrate how tissue CE emerges from cellular behavior. Cellular Potts models (CPM, see Box 1) show that tensional forces generated between cells by filopodia extensions can drive CE in a robust way [44]. It was shown that these filopodia-generated intercalations can help explain limb bud elongation when cells are oriented towards the nearest ectoderm [36]. Moreover, cell intercalations perpendicular to the PD axis generate fast and robust limb elongation, when combined with high cell proliferation at the distal end [36,45]. Interestingly, vertex models of cell intercalation show that the addition of directional cell division creates extra robustness to tissue elongation [45].

### 3.4. Ectodermal Molding

The elongation of the limb bud occurs under the mechanical constraint provided by the ectoderm. Lau et al. found that the ectoderm is anisotropically stressed by mesodermal growth due to the initial limb geometry [46]. This polarizes ectodermal cells along the DV-axis and thereby directs ectodermal remodeling [46]. As the limb bud is formed, the cells gradually become oriented along the proximodistal axis. Tensile stress induced by micro-pipette aspiration revealed that anisotropic stress directs division orientation as well as rosette resolution of ectodermal cells (Figure 3A) [47], while tetrad and rosette formation require cell intrinsic forces [48]. Furthermore, the ectoderm actively enhances this stress through intercalations in the DV direction. These cell rearrangements are directed by the principal stress component, thereby regulating limb elongation [49]. More specifically, in the case of isotropic growth of the underlying mesoderm, a flattened elongated limb bud can still be obtained when the ectoderm shrinks relatively in the direction of the principal stress [49].

### 3.5. Tail Bud Elongation vs. Limb Bud Elongation

Tail bud elongation is a different model system often used to research morphogenetic processes. The tailbud is the growing posterior end of the embryo and has been extensively studied in zebrafish and chick. Many similarities can be seen between the tail bud and the limb bud. For both systems, FGF and WNT are known to be crucial morphogens. Additionally, in both cases the ECM is primarily made up of fibronectin and a stiffness gradient is observed throughout the tissue [50]. Although both model systems seem similar some differences can be noted. The tailbud elongates unidirectionally whereas the limb bud becomes flattened and widens at the growing end. This geometrical difference also affects the FGF signaling center which is stretched along the apical ectodermal ridge in the limb bud, creating a more radial gradient compared to the linear gradient in the tail bud (Figure 3). Despite these differences, insights from tailbud elongation could prove useful for limb bud research.

Tail elongation in both chicken and zebrafish embryos involves a cell motility gradient controlled by FGF (Figure 3B) [25,51]. This motility gradient is accompanied by an opposing gradient in cell density [25,51]. It has been suggested that the cell motility gradient induces this density gradient, thereby driving tail elongation at short timescales by tissue level volume increase [25]. Yet, at long timescales, cell injection from the tail bud to the elongating tail is needed to sustain elongation [25]. In zebrafish tail elongation, these changes in cell motility and tissue density were linked to the unjamming transition [51,52]. At the posterior end, the tissue exhibits fluid-like behavior, enabling tissue shaping, while at the anterior end, the tissue appears solid, maintaining tissue shape and providing support for the growing tail [51]. A similar mechanism could be present for limb bud elongation since both the FGF- and cell motility gradient have been observed [25,26,30,36,51]. However, it is unclear to what extent this unjamming transition complements elongation in the limb bud. The proximal part of the limb bud is not truly jammed as cell rearrangements remain present [36]. Moreover, cell intercalations have been found to fluidize tissue [45]. Perhaps the central condensations could provide enough support for the elongation?

## 4. Mechanisms of Condensation

Pre-cartilage condensations, forming in the third stage of the limb bud development, initiate the formation of skeletal elements in the limb bud. These condensations can be visually distinguished from flanking mesenchyme due to changes in ECM and cell density [53] resulting in reduced opacity. During condensation, the existing ECM is partially removed, and new ECM components are secreted. Density measured in a chicken forelimb at stage HH24 has been found to increase from 13.44 cells/1000 µm${}^{2}$ to 19.07 cells/1000 µm${}^{2}$ [54]. Another study found that cells in axial areas increased from 13.80 cells/1000 µm${}^{2}$ to 27.34 cells/1000 µm${}^{2}$ between HH20 and HH26. On the other hand, density in peripheral regions of these limb buds only increased from 14.69 cells/1000 µm${}^{2}$ to 19.47 cells/1000 µm${}^{2}$ [55]. It is important to note that these measurements can be influenced by fixation techniques which could explain the discrepancy between both experiments.

The origin of this density increase has been the subject of intense research. At a chemical level, many important elements have been uncovered [56], including a reaction-diffusion system with a key role for transcription factor SOX9 and morphogens BMP, WNT and FGF [5,57]. However, the mechanical regulation of this process is still unclear. At first, increased cell proliferation was suggested to explain the increased density. However, proliferation in proximal condensing regions was found to be slightly lower as compared to distal regions [14]. A second proposed mechanism for the density increase involves migration of cells into the condensing region, leading to deformation of the ECM. These local compressions lead to a higher density of adhesion molecules creating a positive feedback loop to cell migration [58]. However, this hypothesis was later rejected since no directed cell migration could be observed towards condensed regions [59].

### 4.1. ECM

**Figure 4:**
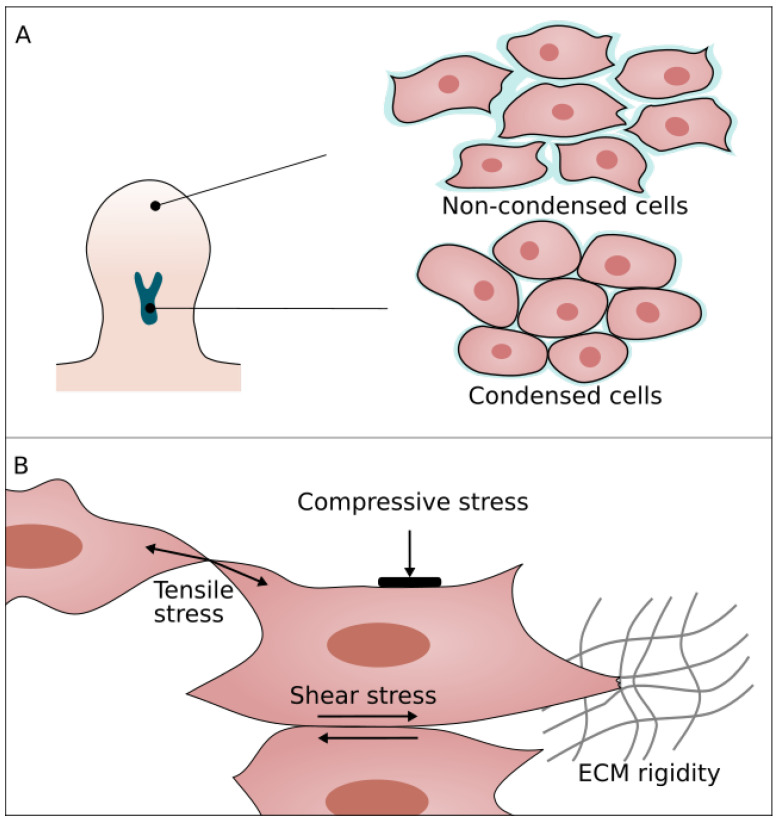
(**A**) During condensation mesenchymal cells become less active and rounded. Additionally, the pericellular matrix, shown in blue, reduces in size allowing more cell–cell contact. (**B**) Mechanical cues influencing mesenchymal cells: tensile stress, compressive stress, shear stress and matrix stiffness.

Prior to condensation, the loose and permeable ECM is primarily made up of fibrillin [53] which is then replaced by other components. An important ECM component during condensation is Hyaluronic acid (HA) [60,61,62,63,64]. HA synthase (has2) is downregulated in the central proximal core of the limb bud [65] simultaneously with expression of CD44. CD44 is a cell surface receptor of HA and regulates HA degradation and endocytosis [66,67,68]. Overall, this causes a reduction of HA in the condensing regions resulting in reduced cell activity and a collapse of inter-cellular space, due to the removal of voluminous HA molecules (Figure 4A) [60,69].

A second major ECM component expressed during condensation is fibronectin (FN) [70,71]. As opposed to HA, FN is upregulated in the proximal central region prior and during the condensation [63,71,72]. This upregulation creates a stiffness gradient in the limb bud [73] which likely mediates durotaxis or haptotaxis [74]. Unfortunately, since no in vivo evidence was found showing active migration towards condensation, it is unclear how this might work. Furthermore, it is possible that the increase in fibronectin-integrin interactions trigger a cascade towards differentiation [75].

It was hypothesized that fibronectin and HA act as a pericellular matrix (PCM) around the cells (Figure 4A. ECM shown in blue) [76]. This has several implications for the condensation process and limb bud morphogenesis. Cellular interactions can be categorized in PCM-mediated interactions and direct cell–cell interactions. PCM-mediated interactions are established through polymer chain cross-links resulting in strong adhesion compared to the N-cadherin and N-cam mediated cell interactions. Forces generated in these PCM interactions are higher than forces generated by cortex contraction following active protrusions, thus aiding cell motility [76]. Secondly, the PCM inhibits N-cam and N-cadherin cell–cell interactions, thereby inhibiting cell differentiation [77]. Additionally, lower adhesion due to a shift from PCM to cell-mediated contact and a lower motility can cause a jamming transition. The shift from PCM mediated interactions to cell–cell interactions is also partly regulated by PG-M. PG-M has inhibitory effects on cell-matrix interactions and is found to be up-regulated in pre-chondrogenic regions [61].

### 4.2. Cellular Properties and Mechanotransduction

During condensation, some distinct changes in cell mechanical properties, such as activity, stiffness or adhesion, take place. These changes are likely regulated by a BMP, SOX9 and WNT reaction-diffusion system [5]. Experiments involving SOX9 mutant mouse chimeras illustrate a possible role for SOX9 in regulating cell mechanics. No visual difference can be observed between SOX9^−/−^- and SOX9^+/+^- cells until condensations are formed. However, during condensation, the SOX9^−/−^-cells are unable to take part in the process and become segregated from the SOX9^+/+^- cells [78]. In micro-aggergate experiments with both cell types, SOX9^−/−^-cells initially take part in the tissue compaction but they are unable to maintain a rounded morphology and are subsequently excluded from condensation [79]. These experiments point to two important mechanical changes. Firstly, the rounded shape and absence of filopodia of condensed cells indicate that they have a lower activity and an increased cortical tension. Secondly, the exclusion of SOX9^−/−^-cells could signify changes in cell–cell adhesion. Indeed, adhesion molecules N-cam and N-cadherin are upregulated by SOX9. Furthermore, these adhesion molecules are also regulated by retinoic acid (RA) which is responsible for sustaining N-cam production until chondrogenesis [80,81].

Morphogens are not the only factors controlling cell and ECM properties. Cells also respond to mechanical changes in their environment [82,83,84,85]. Through mechanosensing, cells can perceive different types of mechanical cues, as illustrated in Figure 4B. These cues can induce cell fate changes or trigger signaling pathways. The importance of mechanosensing is demonstrated in micromass assays using pre-condensation limb bud cells. In these micromass experiments, cells were geometrically constrained in microchannels and allowed to condense. Condensations formed in more narrow channels were found to express more SOX9. Thus, tighter geometric constraints promote condensation. Furthermore, it was found that cells express more SOX9 when stressed by a contracting substrate or cyclic mechanical loading [86,87,88,89].

One of the proposed mechanosensing mechanisms in the limb bud is based on RhoA/ROCK activity. The RhoA/ROCK pathway regulates the actin cytoskeleton. RhoA enhances the formation of focal adhesions and stimulates ROCK. In turn, ROCK increases actomyosin contractility and thereby cellular tension. RhoA/ROCK is activated through different forms of cellular stress; however, small differences in the pathway can occur depending on the type of stress. For instance, tensile stress and matrix stiffness both upregulate RhoA and ROCK [90,91], whereas compressive stress only activates RhoA [92]. This illustrates the importance of quantifying the mechanical environment during limb bud formation as this will influence the type of stress that the cells experience.

The RhoA/ROCK pathway not only regulates cortical tension and focal adhesion, but it also influences chemical signaling and transcription. For example, inhibition of RhoA causes rounding of cells as well as enhanced SOX9 expression [93,94]. Conversely, upregulation of RhoA prevents condensation [81,95]. Additionally, inhibition of ROCK in the pharyngeal arch was found to inhibit condensations [96]. To suggest how these processes might affect the limb bud we can consider the stiffness gradient observed in the limb bud [73]. The stiffer ECM near the middle of the limb bud can trigger the RhoA/ROCK pathway in a specific way causing the cells to condense. However, during condensation, cell shape changes or new cell contacts can further influence the mechanical response of the cells. This way each cell encounters a complex set of mechanical cues which will ultimately decide its fate [97,98]. Current experimental methods cannot comprehensively quantify all the different mechanical cues that cells experience. Here, computational models may help to map different stress throughout the tissue under different conditions. Recently, such maps have been created for zebra fish tail bud axis-elongation using a Finite Element Model (FEM, see Box 1) [52]

The concept of how mechanosensing can complement expression of morphogens has been explored in a mathematical model by Mercker et al. In their FEM model, morphogens locally induce tissue curvature which in turn positively feeds back morphogen expression [99,100]. This mechanism is supported by an epithelial curve sensing mechanism; deformation of the epithelium results in an increased apical-to-basal cell area ratio that can translate to a biochemical signaling cascade [58]. Furthermore, bulging of the underlying mesoderm will eventually lead to a local increase in biochemical signaling molecules [58]. This mechanism can complement Turing patterns of mesenchymal condensation.

## 5. Conclusions

Recent years have seen remarkable advances in understanding the biomechanical and mechanobiological processes that regulate early limb bud development. However, a complete picture of the developmental process remains absent. Although the process is presented here as three distinct steps, it stands to reason that these stages overlap in time. Furthermore, the contribution of each possible mechanism as well as its timing remain unclear. To obtain a more profound understanding of the workings and timing of the different mechanical elements, the mechanical properties of cells and tissue need to be mapped throughout the tissue over several stages of limb development.

Furthermore, the role of mechanotransduction during the different stages of development is incompletely understood. In vitro experiments illustrated the biochemical response of cells to different mechanical cues. These responses can differ depending on the applied mechanical cue which can in turn alter the mechanical response of the individual cells. This further highlights the need to quantify cellular and tissue mechanics in vivo during subsequent stages, since different mechanisms will trigger different cell responses further down the line. This is no small feat, as it will require reliable experimental techniques in combination with advanced computational and mathematical modeling. However, with current advances in high resolution in situ spatiotemporal microscopy techniques, novel mechanical characterization methods and high-performance computing (see Box 1 and Box 2), this ambitious goal might enter the realm of feasibility in the coming years.

## 6. Box 1: Computational Models of Tissue and Cell Mechanics in the Limb Bud

**Figure 5:**
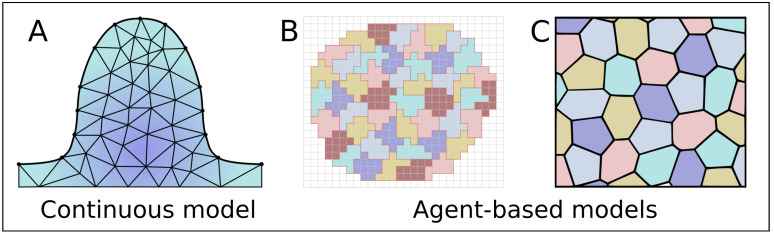
Computational models used to investigate limb bud development. (**A**) Continuous grid-based models, (**B**) Cellular Potts Models and (**C**) Vertex models.

In 1969, Ede and Law created one of the first computational simulations of limb bud elongation. Their lattice-based model with simple rules only considered cell proliferation and movement. With this model they evaluated whether graded proliferation could explain outgrowth. Even though their findings have been refuted, they illustrated the potential of computational modeling. Since then, many diverse types of models have been created. Continuous, partial differential Equation (PDE) models (Figure 5A) are often used to simulate the limb bud. Among these, Finite Element Models (FEM) as created by Dillon and Otmer are often applied to solve PDEs. In FEM models, the mesenchyme is modeled as a viscous fluid surrounded by elastic boundaries representing the ectoderm. The movement of the tissue is calculated with modified versions of the Navier–Stokes Equation [101] or simply the Stokes Equation [14,102]. Cell division in these models is represented as a volume increase of the fluid [103].

A major benefit of these continuum models is that they can be easily combined with existing models of morphogen diffusion [5,104]. The reaction-diffusion equations can be solved with the mesh representing the mesenchyme. However, little can be learned about individual cell behavior and how it affects development since individual cells are not explicitly simulated. For this reason, other than bulk tissue properties, the effect of proliferation rate is the only cell property that can be assessed with this model. A different type of continuum model exists specifically to investigate ectodermal properties. In these models the ectoderm is simulated as a visco-elastic triangulated shell while mesenchymal growth is represented as a constant pressure [46,49].

Agent-based models represent each cell as an individual entity making it possible to investigate individual cellular properties. The Cellular Potts Model (CPM) (Figure 5B) is an often-used method, in this stochastic grid-based method, a single cell occupies multiple lattice sites. Sites at the boundaries of the cells can change occupation thus moving the boundary. Whether or not a boundary is moved is determined by the free energy which depends on cell properties such as compressibility, surface tension, elasticity and adhesion [36,44,105,106,107]. Similar to FEM models, these models can be combined with a continuum approach to solve the morphogen diffusion throughout the tissue making it possible to simulate graded proliferation [105] and condensation [106]. More recently, an adapted model which included lamellipodia pulling forces was created to simulate convergent extension during limb bud outgrowth [36,44].

Finally, in vertex models (Figure 5C) tissue is represented as a spring-lattice network, each lattice node represents a single cell or a cluster of cells [13]. A different type of nodes on the outer edge represents ectodermal cells. Proliferation can be simulated by adding new nodes to the network. Vertex models with convergent extension and directed division show that Hertwig’s rule complements CE to create robust elongation [45]. However, few vertex models for limb bud development exist due to the difficulty of modeling dynamic boundaries.

The advances made in computational biology show the value of computational models by offering new perspectives and insights. However, these models should be interpreted with care and within their intended use [108]. To this end steps have been taken to create a framework for credibility assessment of computer models, centered around the concepts of verification, validation and uncertainty quantification [109,110]. These developments were driven by regulatory science requirements [111,112] but the resulting quality measures benefit models in all stages of research and development.

## 7. Box 2: Experimental Measuring Techniques

In order to better understand the morphogenetic processes, it is crucial to quantify tissue and cell mechanics in vivo. At cell level, cell–cell and cell-ecm adhesion as well as cortex contraction play a major role in the generation of forces within the tissue.

Atomic force microscopy: AFM is an often-used method for measuring material properties of biological samples. The samples are probed with cantilevers of a known spring constant. By measuring the deformation of the cantilever during indentation an apparent stiffness of the sample can be obtained [113]. Depending on the cantilever spring constant and probe shape, AFM can be used to measure stiffness of individual cells as well as entire tissues. Furthermore cell–cell adhesion can also be measured by attaching one cell to the cantilever and using it to probe other cells or tissues [114]. Measurements are precise and fast but can only probe the surface of samples.

Droplets and magnetic particles: Injection of different types of particles is a novel method for measuring cell generated forces. Micron-sized oil droplets are inserted in the tissue after which anisotropy is measured by evaluating the shape changes of the droplet [115]. Since surface tension of the droplet is known, forces can be estimated. Furthermore, these droplets can be combined with ferrofluids making it possible to apply forces on the tissue [116]. Recently, magnetic micro-particles have been used to probe tissue stiffness. When injected into the tissue and placed in a homogeneous magnetic field, the displacement of the particles yields a 3D stiffness map of the entire tissue [73].

Micropipette aspiration: Using a micropipette, negative pressure is applied on the sample. Depending on the diameter the negative pressure can be applied to a single cell or a tissue. Stiffness can be calculated from the applied pressure and deformation of the sample [117]. Alternatively, two cells can be captured in a dual micropipette setup. By analyzing cell shape when pushing and pulling cells together, adhesion energy and cortex stiffness can be estimated. Micropipette aspiration experiments are relatively cheap and simple; however, large deformations of the tissue are needed and accuracy is limited by optical imaging [114].

## Data Availability

Not applicable.

## Abbreviations

The following abbreviations are used in this manuscript:

PI	Positional information
RD	Reaction diffusion
EMT	Epithelial-to-mesenchymal transition
DAH	Differential adhesion hypothesis
DITH	Differential interfacial tension hypothesis
AER	Apical ectodermal ridge
FEM	Finite element method
PCP	Planar cell polarity
CE	Convergent extension
CPM	Cellular Potts model
HA	Hyaluronic acid
FN	Fibronectin
PCM	Pericellular matrix
RA	Retinoic acid
PDE	Partial differential equation
